# Study Protocol for ‘PsilOCD: A Pharmacological Challenge Study Evaluating the Effects of the 5-HT2A Agonist Psilocybin on the Neurocognitive and Clinical Correlates of Compulsivity’

**DOI:** 10.7759/cureus.78171

**Published:** 2025-01-29

**Authors:** Sorcha O'Connor, Kate Godfrey, Sara Reed, Joseph Peill, Cyrus Rohani-Shukla, Mairead Healy, Trevor Robbins, Ana Frota Lisboa Pereira de Souza, Robin Tyacke, Maria Papasyrou, Dea Stenbæk, Pedro Castro-Rodrigues, Martina Chiera, Hakjun Lee, Jonny Martell, Robin Carhart-Harris, Luca Pellegrini, Naomi A Fineberg, David Nutt, David Erritzoe

**Affiliations:** 1 Department of Brain Sciences, Faculty of Medicine, Imperial College London, London, GBR; 2 Department of Brain Sciences, Faculty of Medicine, Imperial College Lonson, London, GBR; 3 Department of Psychology, University of Cambridge, Cambridge, GBR; 4 Department of Clinical, Pharmaceutical and Biological Science, University of Hertfordshire, Hatfield, GBR; 5 Department of Psychology, University of Copenhagen, Copenhagen, DNK; 6 Neuropsychiatry Unit, Champalimaud Foundation, Lisbon, PRT; 7 Department of Biomedical and Neuromotor Sciences, University of Bologna, Bologna, ITA; 8 Neurology, University of California, San Francisco, USA; 9 Department of Medicine, Surgery and Health Sciences, University of Trieste, Trieste, ITA; 10 Department of Mental Health, Psychiatric Clinic, Azienda Sanitaria Universitaria Giuliano-Isontina (ASUGI), Trieste, ITA; 11 School of Life and Medical Sciences, University of Hertfordshire, Hatfield, GBR; 12 General Adult Psychiatry, Hertfordshire Partnership University NHS Foundation Trust, Hertfordshire, GBR; 13 Department of Psychiatry, Cambridge University Clinical Medical School, Cambridge, GBR

**Keywords:** 5ht2a, cognitive, disorder, flexibility, obsessive-compulsive, obsessive-compulsive disorder (ocd), psilocybin, psychedelics, symptoms, therapy

## Abstract

Background: Obsessive-compulsive disorder (OCD) is a complex condition marked by persistent distressing thoughts and repetitive behaviours. Despite its prevalence, the mechanisms behind OCD remain elusive, and current treatments are limited. This protocol outlines an investigative study for individuals with OCD, exploring the potential of psilocybin to improve key components of cognition implicated in the disorder. The PsilOCD study strives to assess the effects of low-moderate psilocybin treatment (10 mg) alongside non-interventional therapy on several facets of OCD. The main focus points of PsilOCD are cognitive flexibility, measured with cognitive tests, and neuroplasticity, assessed through electroencephalography (EEG).

Methods: 20 blinded participants with OCD will complete two dosing sessions, separated by four weeks, where they will receive 1 mg of psilocybin on the first and 10 mg on the second. The first dose serves as an active placebo, and the latter is a low-moderate dose that induces relatively mild-moderate emotional and perceptual effects. Participants will be supported by trained psychedelic therapists, who will sit with them during each dosing session and provide virtual preparation and integration sessions over the 12-week study period. Therapeutic support will be the same for both the 1 mg and 10 mg sessions. PsilOCD’s primary outcomes include scores in the intradimensional-extradimensional (ID-ED) shift task, which is an established measure of cognitive flexibility, and neuroplasticity as quantified by a visual long-term potentiation (vLTP) task. This task is delivered as part of an EEG paradigm and measures acute quantified changes in neuroplasticity in the brain’s visual system. The ID-ED task will be conducted twice, two days after each dosing session, and the EEG recordings will also be taken twice, immediately after each session. Secondary outcome assessments will include OCD and affective symptom severity, as well as an array of patient-reported outcome measures (PROMs), in the form of questionnaires designed to assess well-being, dissociable and well-established mood-related (affective) measures, and participants’ subjective experience of the psilocybin experience.

Discussion: This study’s results are expected to offer critical insights into the neural mechanisms underlying the effects of psilocybin-assisted therapy in treating OCD, and whether these correlate with changes in the cognitive features of the condition. As a secondary aim, it will ascertain whether a low, tolerable dose is a feasible and efficacious clinical treatment, and will provide crucial data to guide the design of a potential follow-up randomised control trial (RCT).

## Introduction

Obsessive-compulsive disorder (OCD) is a complex psychiatric disorder characterised by persistent, recurrent, and distressing thoughts (obsessions) and repetitive, ritualistic behaviours or mental acts (compulsions) that the individual feels driven to perform in response to these obsessions. The obsessions can pertain to a broad range of themes, such as contamination, order, symmetry, developing psychosis, or harming others [1]. Performing compulsions is an attempted solution to assuage anxiety symptoms, but ultimately keeps sufferers trapped in the maladaptive vicious cycle [2]. Although the exact cause of OCD is not fully understood, research suggests that a combination of genetic, neurobiological, and environmental factors contribute to its development [3].

The cognitive features of OCD

Despite the diverse ways OCD manifests, the presence of obsessions and compulsions distinguishes it from other anxiety disorders that typically involve only avoidance. Therefore, it is likely that OCD involves cognitive factors as well as a genetic predisposition to pathological anxiety. Recent research has shifted away from viewing OCD solely as an anxiety-related condition and has begun to explore it as encompassing various cognitive and emotional features. The Diagnostic and Statistical Manual of Mental Disorders, Fifth Edition (DSM-5) and the International Classification of Diseases, Eleventh Revision (ICD-11) now recognise a category called obsessive-compulsive related disorders (OCRDs), which includes OCD along with other compulsive disorders like body dysmorphic disorder, trichotillomania (hair pulling), and excoriation (skin picking) disorder [4-6].

This shift in understanding redefines OCD beyond solely an anxiety disorder triggered by anxiety-inducing stimuli, emphasising the role of compulsivity. The establishment of OCRDs was driven by pioneering studies in neurobiology, including brain imaging and insights from animal models. These studies identified alterations in neurocognitive mechanisms linked to behavioural inhibition, cognitive inflexibility, reversal learning, and habit formation alongside changes in the underlying frontostriatal neural circuitry. These findings extend to obsessive-compulsive symptoms across a broader spectrum of OCRDs [7,8].

Cognitive inflexibility: an OCD endotype and prospective target for intervention

Cognitive flexibility, the ability to adapt and switch between different conceptual representations, is consistently impaired in individuals with OCD. Meta-analyses of neuropsychological tasks reveal medium-sized effects indicating cognitive inflexibility in patients [9-11]. Cognitive inflexibility has thus been identified as a potential endophenotype in OCD, with studies showing poorer cognitive flexibility in unaffected relatives compared to controls [12]. Deficits in attentional set-shifting abilities have been observed in both patients and relatives, independent of OCD symptom severity and even in remitted patients [13,14]. 

The Wisconsin Card Sorting Test and the Cambridge Neuropsychological Test Automated Battery Intradimensional/Extradimensional (CANTAB ID-ED) Set-Shifting Task are commonly used to assess cognitive flexibility. The ID-ED task, administered using computerised devices, specifically evaluates the ability to generalise learned rules and shift attention to new dimensions [15]. Studies employing the ID-ED task have found deficits in cognitive flexibility, particularly at the extradimensional stage, in individuals with OCD compared to those with trichotillomania [16].

These impairments are associated with reduced functional connectivity between the striatum and the lateral prefrontal cortex (PFC) [17,18]. Research in both marmosets and humans supports the relevance of this brain circuit for cognitive flexibility [19,20]. Furthermore, deficits in cognitive inflexibility extend to the ability to adapt behaviour after negative feedback, as seen in tasks like reversal learning and delayed alternation. Patients with OCD and their unaffected relatives exhibit deficits compared to controls, with relatives showing milder impairments [21]. 

Electroencephalogram (EEG) studies indicate frontal deficits, consonant with these findings. One study by Lee et al. (2023) specifically observed increased theta activity in the anterior cingulate cortex (ACC) of OCD patients, which was not seen in patients with panic disorder. It may be a compensatory mechanism of the brain to overcome the OCD symptomatology, and is consistent with previous observations linking heightened frontal theta activity with cognitive and affective dysregulation in OCD [22,23]. This also aligns with findings by Perera et al., who reported increases in both delta and theta power in the fronto-temporal regions of OCD patients, indicating significant alterations in resting-state brain activity compared to healthy controls [24]. Although enhanced oscillatory power in lower-frequency bands might be a meaningful OCD correlate, it is unclear how closely this correlates with clinical and cognitive symptoms in real time [24].

However, Kamaradova et al. found that EEG correlates elicited in OCD sufferers differed in response to a script designed to depict a generic anxiety-inducing situation versus an autobiographical scenario concerning their specific fears [25]. In OCD patients and not controls, the autobiographical script elicited increased ‘high beta’ activity (20.5-28 Hz) in the left frontal lobe and limbic lobes, unlike the general anxiety script. The strongest source was in Brodmann area 10 (the anterior-most part of the human PFC). Tellingly, these regions form part of the cortico-striatal-thalamo-cortical (CSTC) circuit observed to be dysregulated in OCD [25].

Recent evidence for altered EEG signals in OCD highlights the presence of an enhanced error-related negativity (ERN) signal, which is now firmly established as an endophenotype of the disorder. This heightened deflection may reflect an amplified response to errors, an excessive demand for cognitive control, or intensified behavioural monitoring in the face of perceived threats. However, a direct relationship between clinical symptoms and these neurophysiological markers has yet to be firmly established [26].

Pharmacological treatments

Up until the 1990s, the tricyclic antidepressant clomipramine was the most commonly prescribed drug for OCD. Clomipramine's effects are primarily attributed to its potent serotonin reuptake inhibition, which leads to increased serotonin levels in the synaptic cleft, thereby enhancing serotonergic neurotransmission [27]. Nowadays, it has been predominantly supplanted by selective serotonin reuptake inhibitors (SSRIs), particularly fluoxetine, fluvoxamine, and sertraline, which also increase synaptic serotonin concentrations. These medications have a more favourable side effect profile and are better tolerated by patients [7,28]. 

Acknowledging the varied definitions of response criteria found in the literature, a recent review indicates that only up to 50% of OCD patients exhibit a response to SSRIs [29]. Adjunctive medications including clomipramine, antipsychotics, and mood stabilisers may be prescribed alongside them, with the addition of antipsychotics such as risperidone or aripiprazole leading to the most significant further reductions of symptoms. However, around 30-40% of OCD patients still remain treatment-resistant, underlying the need for new alternative treatment approaches [7].

Recent studies have shown that certain stimulants can also improve OCD symptoms, at least in the short term; there is preliminary evidence suggesting caffeine and dextroamphetamine can improve OCD symptoms individually, as well as nicotine [30-32]. These stimulants enhance dopaminergic and noradrenergic transmission in the PFC, leading to improved executive functions and attention. In the basal ganglia, they increase dopamine levels, thereby impacting motor control and reward processing. These changes result in altered activity within the CSTC circuit, which is crucial for regulating cognitive and behavioural responses [33]. By engaging goal-oriented faculties, temporarily distracting sufferers from their OCD-related thought patterns [34,35]. Despite a range of pharmacological treatments moderately alleviating OCD symptoms, there is a clear need for better interventions that more precisely target the underpinnings of the condition and achieve higher remission rates.

Psychological intervention

Currently, cognitive behavioural therapy (CBT), particularly exposure and response prevention (ERP) therapy, is the first line and most effective treatment for OCD [36,37]. ERP involves exposing patients to their feared stimuli while encouraging them to resist performing compulsive behaviours. This modality allows them to test their ‘worst outcome’ hypothesis and learn that their fears are not valid threats [9]. Traditionally, the efficacy of ERP was attributed to its ability to deconstruct fear-based associations via habituation. However, modern neuroscience suggests that these associations are suppressed, rather than broken [38].

Influenced by translational neuroscience research in animals and healthy volunteers focused on fear extinction, Craske et al. posit that this ‘inhibitory learning’ underpins successful exposure therapy [39]. This involves the encoding and long-term retention of newly learned, non-threatening associations to suppress existing fear memory engrams [39]. Mechanistically, it appears that the amygdala and the medial prefrontal cortex (mPFC) play a key role in this active learning process [34]. Inhibitory neurons in the mPFC release gamma-aminobutyric acid (GABA), which inhibits fear memories encoded in the amygdala [38]. However, despite interactions between the mPFC and the amygdala being associated with the fear extinction process in both rodents and humans, it is unclear whether this underpins ERP [36].

Could psychedelics represent a novel targeted OCD intervention?

Recent research suggests that psychedelic compounds might offer a novel treatment avenue in psychiatry, and show promise to target more than one facet of OCD. Over the past 10 years, there has been a remarkable resurgence in the field of clinical research involving psychedelic substances. Among these classic psychedelics are lysergic acid diethylamide (LSD), psilocybin (the active component found in ‘magic mushrooms’), and dimethyltryptamine (DMT), the active ingredient in ayahuasca. An increasingly large body of evidence recognises these psychedelic compounds for their safety and effectiveness in various clinical contexts. These contexts include the treatment of conditions such as depression, and end-of-life distress using psilocybin-assisted therapy [40,41]. Additionally, psilocybin has shown promise in benefiting the general well-being of individuals in good health [42,43].

Psilocybin, and other classic psychedelics, produce substantial alterations in consciousness, eliciting complex visual imagery at high doses, alterations to affect often experienced as ‘emotional breakthroughs’, and changes in sensory perception [44,45]. These subjective effects largely stem from their strong affinity for the serotonin 5-HT2A receptor, which is highly expressed in the PFC. When psilocybin interacts with these receptors in the PFC, it dysregulates activity in the cortex, impacting networks such as the default mode network (DMN), a brain network including the mPFC, posterior cingulate cortex (PCC), and temporal-parietal junction (TPJ) [46]. Studies by Erritzoe et al. and Carhart-Harris et al. show that psychedelics, including psilocybin, dysregulate cortical activity, impacting self-focused thoughts and altering the processing of external stimuli [47,48]. 

Activation of serotonin 5-HT2A receptors appears to dysregulate population-level spontaneous neural oscillations, and may subsequently account for increases in markers of anatomical neuroplasticity including brain-derived neurotrophic factor (BDNF) [9,50]. BDNF is a protein crucial for the survival, growth, and maintenance of neurons. It plays a vital role in neuroplasticity, which is essential for learning and memory. Psilocybin seems to augment the efficacy of BDNF, potentially by functioning as a ligand for its receptor TrkB and elevating BDNF levels directly. These alterations collectively result in heightened activity at the TrkB receptor, which governs a molecular process known as long-term potentiation (LTP). LTP serves as the principal mechanism underlying neuroplasticity and forms the basis for both conscious and implicit learning mechanisms [51]. In the context of psychedelic-assisted therapy, increased plasticity via 5-HT2A agonism may increase an individual’s sensitivity to OCD-specific psychotherapy like ERP.

Early evidence pointing to psilocybin’s potential in treating OCD

In a therapeutic context, psychedelics are hypothesised to ‘open a window’ of psychological plasticity that facilitates the reappraisal of rigidly entrenched, maladaptive thought patterns towards therapeutically beneficial outcomes [52,53]. Such increases in psychological flexibility make OCD a desirable target for investigation due to its hallmark psychological rigidity [7,8]. Psilocybin research in the context of OCD is only in its early stages. The most promising investigation was conducted by Moreno et al. in 2006; they found that administering different controlled doses of psilocybin (0.1-0.3 mg/kg) in a non-placebo study led to a significant reduction in OCD symptoms among treatment-resistant patients who had not responded to typical treatments like SSRIs [54].

Since then, a 2022 case report found that a treatment-refractory patient with ‘severe’ OCD (as deemed by Yale-Brown Obsessive Compulsive Scale (Y-BOCS) score) achieved subclinical symptoms for 12 weeks following the dose [55]. In line with this, two case studies in 1987 and 1997 reported LSD and psilocybin to be helpful [56,57]. In the former study, a teenager with severe OCD found acute relief from moderate doses of both psychedelics. The latter involved a man with similarly severe symptoms taking an unspecified dose of psilocybin-containing mushrooms daily for four years. He experienced a significant decrease in his symptoms, which persisted for two years after he stopped his dosing schedule. However, his symptoms returned after this period.

In the most recent study by Moreno et al., participants saw a notable drop in their Y-BOCS scores, and adverse reactions or heightened anxiety related to the psilocybin were not reported. These findings suggest that the therapeutic potential of low doses of psilocybin for OCD may be linked to activating the 5-HT2A receptor within the relevant brain circuitry. Notably, the three highest doses all yielded significant improvements, implying that a low dose of psilocybin (10 mg, comparable to the lower 0.1 and 0.2 mg/kg doses administered by Moreno et al.) may be a viable treatment [54]. This is useful to determine, since many OCD patients fear loss of control and may be opposed to having a more intense psychedelic experience. Most studies assessing psilocybin in the context of mental health have explored 25 mg doses [48,58]. However, if meaningful improvements in OCD symptoms can arise from the administration of 10 mg, this may be preferable to many patients.

Literature suggests that 1 mg of psilocybin serves as a good control, or active placebo [40,42,48]. This dose falls at the lower limit of what is typically regarded as a 'microdose', and does not produce noticeable physiological, perceptual, or emotional effects [43,59]. Importantly, opting for a 1 mg dose rather than an inert placebo also enables better blinding, as all patients can be told that they will be given ‘up to 10 mg’ of psilocybin during each session. This decreases the chance of a ‘disappointment effect’ from the 1 mg dose, mitigating the risk of participants believing that they have been given no psilocybin during the first dosing session and responding emotionally. Participants should be given the same therapeutic support before, after, and during both dosing days.

How psilocybin might improve the nuanced, dissociable cognitive deficits of OCD

Apart from the one conducted by Moreno et al., there has been a paucity of OCD-focused psychedelic studies, but considering the molecular effects of psilocybin alongside the cognitive features of OCD suggests it could be a valuable intervention for patients. Recent research by Torrado-Pacheco et al. demonstrated that psilocybin acutely improved set-switching in rats, a key aspect assessed in the ID-ED cognitive flexibility task [60]. In humans, the therapeutic effects of psychedelics in treating major depressive disorder involve reducing rumination and enhancing self-perception, leading to a broadening of one's perspective on the world. This enhancement in openness most likely involves increases in cognitive flexibility, reinforcing the rationale for the proposed utility of psilocybin in increasing flexibility at the neural and psychological levels [61-65].

Along with showing deficits in cognitive flexibility, OCD patients exhibit altered decision-making. Decision-making involves a trade-off between certainty and time, and OCD patients often spend excessive time deliberating minor decisions. They show reduced coupling between action and confidence, leading to poorer decision-making, possibly due to excessive uncertainty about environmental transitions [18]. This unnecessary information-seeking is likely attributable to impaired metacognition, reflected in low self-confidence, pessimistic and mistrusting beliefs about others and the world around them, and a consequent unwillingness to make decisions without amassing significant evidence [18,66,67]. The Relaxed Beliefs Under Psychedelics (REBUS) model suggests that psychedelics may alter the weight of high-level priors pertaining to metacognitive processes and one’s perception of the world around them, potentially improving decision-making efficiency [68]. 

Offering a fresh yet complementary perspective on OCD, compulsions can be likened to habits, contributing to unevenness in goal-oriented behaviour. Brain circuitry, particularly frontostriatal loops involving regions like the caudate, putamen, and orbitofrontal cortex, plays a significant role in both OCD and habit formation [69]. Experiential learning studies also reveal that OCD patients are biased towards habit formation over goal-directed thinking, which correlates with increased activation of the caudate nucleus compared to controls [70,71]. Considering these findings collectively, psilocybin could reduce habitual compulsions and improve confidence self-evaluation by acting on OCD-implicated areas within the CSTC loop.

Overview and aims

This paper presents the protocol for the mechanistic study ‘PsilOCD’, which investigates the effects of psilocybin therapy (PT) in an OCD population. The principal focus of this trial is the impact of psilocybin on cognitive flexibility and neuroplasticity, primarily captured by cognitive tasks conducted in the days following its administration and EEG recordings immediately after the psychedelic state. Secondary outcomes will aim to capture changes in OCD symptomatology and related affective and personality-related measures through clinician-conducted scales and psychological questionnaires (classed as patient-reported outcome measures (PROMs)).

The impact of psilocybin on neuroplasticity will be further explored through blood analysis for BDNF, since this biomarker has been linked to psychedelic use. In an exploratory fashion, plasma levels of established inflammatory modulators will also be assessed. Although not a key outcome, this may shed light on whether psilocybin modulates the immune system, which is believed to be implicated in psychiatric conditions including OCD. As a feasibility study of up to 20 completers, PsilOCD aims to represent a preliminary, yet influential, investigation into the potential impact of psilocybin on this patient population. By publishing this study protocol, we hope to contribute towards a scientific culture of transparency and rigour.

## Materials and methods

Our primary outcomes investigate a) cognitive features of OCD through the CANTAB intradimensional-extradimensional (ID-ED) task, which assesses attentional ‘set-shifting’ and b) neuroplasticity through the visual long-term potentiation (vLTP) EEG paradigm. This feature of cognition is impaired in OCD and unaffected relatives, framing it as a potential cognitive endophenotype. OCD symptoms will also be measured using the Y-BOCS, and depression scores - accounted for by the Montgomery-Asberg Depression Rating Scale (MADRS) - will also be collected. Further investigations will include self-reported psychological questionnaires designed to assess participants’ affective symptoms, including measures of anxiety, as well as their perception of the subjective psilocybin experience. The complete list of outcomes is presented in the 'outcome measures' section.

Recruitment

We will recruit up to 20 completing participants with OCD as defined by the DSM-5 criteria (assessed using the Mini-International Neuropsychiatric Interview (MINI)). Study completion is considered the fulfilment of the final study visit. Recruitment will occur via flyers, word-of-mouth, and from a pool of self-referrals submitted via a secure centralised e-mail address using a standardised referral form. Summarised participant information sheets will be openly accessible on our study website. Primary care providers will be contacted to confirm eligibility. Full entry criteria are outlined in Table 1 below.

**Table 1:** Key PsilOCD inclusion and exclusion criteria *Antipsychotic medications may attenuate the effects of psilocybin, and limited available evidence suggests that mood stabilisers like lithium may interact with psilocybin and lead to untoward side effects [72,73]. In this population, people may be using these medications as adjunctive treatments alongside SSRIs. OCD: Obsessive-compulsive disorder; SSRI: Selective serotonin reuptake inhibitor; ERP: Exposure-response prevention

Category	Inclusion Criteria	Exclusion Criteria
Demographics	- Aged 20 to 65 years; - Any gender	- People who are pregnant, planning to get pregnant, or currently breastfeeding
Mental Health	- Primary diagnosis of OCD; - Experienced OCD for at least 12 months	- Current or previously diagnosed psychotic disorder, bipolar disorder, or mania; - Immediate family member with a diagnosed psychotic disorder; - History of serious suicide attempts (requiring hospitalisation)
Physical Health	- Stable physical health; - Able to engage in the physical demands of dosing session	- Unstable physical illness; - Significantly abnormal clinical test result; - Heavy smoker and/or unable to complete the dosing session without a smoking break
Willingness and Compliance	- Willing to comply with protocol; - Able to identify a trusted person for emergency contact; - Excellent understanding of English (for questionnaires); - Ambulant and capable of attending outpatient visits; - Comfortable using a computer and accessing the internet from home; - Willing to attend some study visits virtually	- Unlikely to comply with the study protocol and lifestyle restrictions; - Unwilling to allow GP or mental health practitioners to be informed of participation or allow the study team access to Summary Care Record; - Unable to engage with physical demands of the dosing sessions (i.e., attend the centre and remain in the research facility for an extended period)
Medication/Treatment	- Not currently using medication that could interact with psilocybin (particularly antipsychotics and mood stabilisers)*	- Currently using medication that could interact with psilocybin (e.g. antipsychotics/mood stabilisers)*; - Currently engaging in any form of OCD-targeted psychotherapy e.g., ERP

Screening and consent 

Eligible self-referrals will be invited to a remote screening call, where initial informed consent will be collected. These screening calls conducted by an OCD-experienced psychiatrist serve to inform prospective participants of the study demands and determine initial eligibility, including a detailed MINI assessment.

If the participant passes this initial eligibility check, they are invited to an in-person screening visit held at the study clinic. Following a thorough explanation of the study and screening process, patients will have the opportunity to ask questions before providing full written informed consent to participate in the PsilOCD study. They will then have a physical exam, further psychiatric assessment, an electrocardiogram (ECG) and a urine drug screening, and blood samples will be taken for routine blood testing. Baseline EEG resting state measures will be collected, allowing for the assessment of EEG tolerability.

Information regarding the screening/enrollment process, including retention and demographics, will be published upon study completion.

Study visits

**Figure 1:**
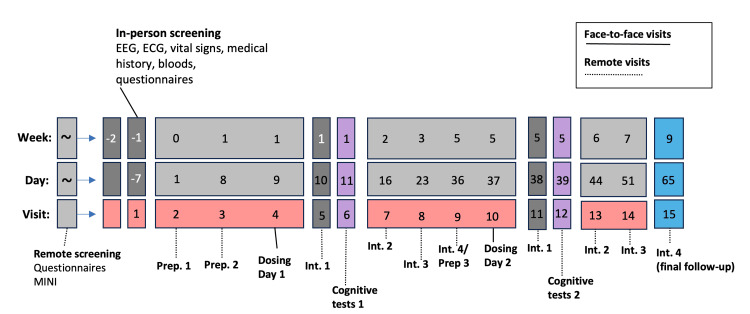
A depiction of the PsilOCD study timeline: the total period from the first preparation session (Prep. 1) to the final integration session (Int. 4). EEG: Electroencephalography; ECG: Electrocardiogram; MINI: Mini-International Neuropsychiatric Interview

Participants will complete study activities at 13 time points over the 12-week study period after screening, of which two are in-person dosing days (see Figure 1 for the schedule). Two trained therapists (or ‘guides’) will accompany participants and provide psychotherapeutic support throughout the duration of the study (more information in the section below). Participants will attend two dosing days and receive 1 mg of psilocybin on the first (active placebo) and 10 mg on the second. Dosing sessions are separated by at least four weeks, each preceded by at least one preparation session and followed by four integration sessions, with the final integration session coming four weeks after the final dosing session.

Preparation

Preparatory sessions serve to initiate the self-reflective therapeutic process, build trust and rapport with the therapists, remind participants of what to expect from the dosing days, and start discussing themes that arise for them related to their OCD symptoms. Any changes in participants’ symptoms, well-being and attitude towards the study are documented. Preparatory sessions occur one week and one day before each Dosing Day.

Dosing Day 1 and 2

The participant will arrive at the clinic (Central and North West London (CNWL)-Imperial Psychopharmacology & Psychedelic Research (CIPPRes) Clinic, at CNWL NHS Trust’s St Charles Hospital) in the morning, and will be administered psilocybin (1 mg on the first visit and 10 mg on the second). The therapists will offer non-directive psychological support throughout the dosing days. One hour after the substance wears off, the EEG vLTP paradigm will be carried out to quantify enhancements in plasticity. Bloods will be taken and tested for BDNF levels, as well as immune markers. The participant’s subjective experience will also be assessed using questionnaires (the Mystical Experience Questionnaire (MEQ), the Challenging Experience Questionnaire (CEQ) and the Emotional Breakthrough Inventory (EBI)), and they will be prompted to estimate the dose of psilocybin that they received. This day will end at approximately 17:30, at which point they will be driven home in a taxi or collected by a trusted contact.

Integration

The participant will attend three integration sessions with the therapists, designed to help them interpret any insights and apply them to their lives. These sessions will take place one day, one week and two weeks after each dosing day. The Y-BOCS will also be conducted again, as well as the aforementioned measures of affective symptoms.

Neuropsychological Task Performance (CANTAB Test Battery)

Study collaborators from the University of Cambridge will administer the CANTAB test battery remotely two days after each dosing session. This will involve ED-shift outcome measures using ID-ED Set Shift, Probabilistic Reversal Learning, and Paired Associates Learning task (a neutral control task).

One-Month Follow-ups

These two remote sessions will take place one month after each dosing session. The first will mainly be a preparation for the second dose, taking place one day before the second dosing day; the second follow-up will be a final psychological integration session, and a discussion of the participant’s experience partaking in the study. The Y-BOCS and affective symptoms tests will also be conducted a final time.

Low-moderate dose psilocybin administration with psychotherapeutic support

While PsilOCD is primarily a pharmacological challenge study, safely administering this drug in clinical populations requires psychotherapeutic attunement and support [74]. There is currently no standardised protocol for this intervention, but common principles shared across studies will be applied in this investigation such as intention setting, offering psychoeducation about navigating psychedelic experiences, and other non-directive and positive humanistic approaches [75]. Even though most of the literature concerning psilocybin treatment involved higher doses of psilocybin than PsilOCD, this trial's psychotherapeutic model is inspired by it and aims to facilitate a compassionate, self-guided psychological journey [76].

To maintain consistent conditions, participants will receive the same therapeutic support before, after and during both dosing sessions. Participants will attend study sessions in a comfortable, softly lit setting, where they will be encouraged to recline and wear eye masks. Recognising the influence of music on emotional states and potentially therapeutic outcomes during the dosing sessions, playlists have been curated by experienced researchers in the field of psychedelics [77]. Participants will stay introspective during the effects of the drug experience to maximise the non-directive approach and be sensitive to opportunities for psychological change [78]. While the psychotherapeutic container is mostly non-verbal on dosing days, the therapists will ask participants to rate the intensity of the psilocybin every 40 minutes.

Since PsilOCD is a feasibility study designed to pave the way for future research into OCD and psychedelics, no OCD-specific therapy is administered. This will permit the lucid assessment of the pharmacological effects of psilocybin when paired with encouraging, but non-interventional, therapeutic support.

Throughout the study, each participant will be supported by two guides. These guides will assist with the entire process, beginning with virtual preparation sessions to help participants understand and get ready for the experience. During each dosing session, the guides will accompany participants in a private room and will follow up with four integration sessions after each dose. Dosing sessions are scheduled for the day after the second preparation meeting. Each participant will spend roughly nine hours at the research facility, with the drug effects expected to last between four and six hours. While participants are encouraged to use eye masks and headphones, this is optional. EEG recordings will be conducted both before and during the peak drug phase. An on-site medical professional will oversee participant safety throughout, giving approval before participants leave the facility. Those living locally may return home if accompanied by a trusted person, while others are provided with overnight accommodation at the facility.

The first integration session is conducted the morning after the dosing day, allowing participants to meet virtually with their guides to discuss their psilocybin experience. All themes that arise, whether related to OCD or not, will be further examined in the integration sessions held over the four-week follow-up period following each dosing day.

Safety, monitoring, and reporting procedures

Maintaining the safety of participants is the paramount guiding principle that informs all decisions related to the study. Participants will be continuously accompanied by a member of our research team on dosing days, and their departure will be subject to the on-site physician's evaluation of their safety. Additionally, the well-being of participants will be assessed by their two therapists the morning following dosing and in all subsequent integration sessions. Participants will be informed that the study psychiatrist is available 24/7 for emergency calls, should anything untoward occur.

Outcome measures

See Table 2 below for the study outcome measures. These comprise neurophysiological, psychiatric, self-reported, and cognitive metrics.

**Table 2:** Summary of PsilOCD Outcome Measures and Hypotheses ID: Intradimensional; ED: Extradimensional; CANTAB: Cambridge Neuropsychological Test Automated Battery; EEG: Electroencephalography; vLTP: Visual long-term potentiation; Y-BOCS: Yale-Brown Obsessive Compulsive Scale; MADRS: Montgomery-Asberg Depression Rating Scale; PROM: Patient-reported outcome measure; BDNF: Brain-derived neurotrophic factor

Outcome Type	Outcome Measure	Hypothesis
Primary Clinical Outcome	Cognitive Flexibility: ID-ED Shifting (CANTAB)	Participants will show improved scores on the CANTAB ID-ED task two days after the 10 mg psilocybin dose compared to two days after the 1 mg dose.
Primary Neuroimaging Outcome	Neuroplasticity: EEG vLTP	Participants will demonstrate higher capacity for neural plasticity two hours post 10 mg dose compared to the 1 mg dose, as indicated by vLTP in EEG recordings.
Secondary Clinical Outcome	Y-BOCS and MADRS Scores	Y-BOCS scores for obsessions and compulsions are expected to decrease after the 10 mg dose but not after the 1 mg dose. MADRS scores will likely follow a similar pattern.
Secondary Clinical Outcome	Cognitive Inflexibility: Reversal Learning (CANTAB)	Participants will show improved scores in reversal learning two days post 10 mg psilocybin dose, but not two days post 1 mg dose.
Secondary Clinical Outcome	Cognitive Inflexibility: Paired Associates Task (CANTAB, Control Task)	Scores are expected to be comparable after both 1 mg and 10 mg doses, as visuospatial tasks should not be affected by 5-HT2A receptor modulation.
Secondary Clinical Outcome	Cognitive Inflexibility: Information-Seeking Task	If psilocybin reduces the rigidity of high-level priors, confidence will improve, and information-seeking behavior will reduce. Significant effects are expected one day after the 10 mg dose, not the 1 mg dose.
Secondary Clinical Outcome	Self-Reported Well-Being and Affective Measures (PROMs)	Compared to 1 mg, the 10 mg dose is expected to improve anxiety and depression-related symptoms, decrease impulsivity (Barratt Impulsivity Scale), increase openness, and decrease neuroticism (Big Five Inventory).
Secondary Clinical Outcome	Acute Subjective Experiential Measures (PROMs)	The 10 mg psilocybin dose will result in significantly higher scores on questionnaires assessing subjective drug effects compared to the 1 mg dose.
Secondary Clinical Outcome	Blood Analysis: Serum BDNF and Plasma Inflammatory Markers	The 10 mg dose, but not the 1 mg dose, will significantly increase serum BDNF levels and reduce plasma markers of inflammation. This outcome is exploratory and focuses on psilocybin's potential acute anti-inflammatory effects.

Mechanistic neurophysiology outcome measures

In order to ascertain the effect of this low dose of psilocybin on neuroplasticity in an OCD population, the vLTP EEG paradigm will be run at three time points during the course of the study. The first EEG session takes place during the in-person screening day to acquire baseline recordings, while the subsequent two are done on both dosing days after the effects of the drug have worn off (two hours after dosing).

The vLTP paradigm uses tetanising visual stimulation to measure the brain's capacity to undergo neuronal plasticity, specifically in the occipital lobe where we can easily measure evoked responses to external visual stimuli. This vLTP paradigm was developed at the University of Auckland and is now incorporated into several psychedelic trials both at the University of Auckland and Imperial College London. As the vLTP paradigm quantifies neuroplasticity, this will provide valuable insight into whether psilocybin enhances it in OCD patients.

Cognitive tasks

As cognitive inflexibility is a hallmark characteristic of OCD and potentially causal to the onset of symptoms, we are investigating it through several cognitive tasks. The first, the ID-ED task, is delivered as part of the CANTAB; it measures participants’ ability to switch their focus from one dimension of a task (set of stimuli) to another. The reversal-learning task, also part of the CANTAB, assesses their ability to learn to no longer apply a rule when it becomes obsolete. The final CANTAB task, the visuospatial paired-associates task, serves as a control and will indicate the order effect at play. Scores are not expected to be influenced by psilocybin. The information-seeking task developed by Schulz et al. measures participants’ tendency to seek extra information, assessing confidence and decision-making [79].

Patient-reported outcome measures

While the primary interest of the study is in the neural mechanisms involved in psychedelic-mediated action in an OCD population, we will also collect efficacy measures at every time point of the study. PROMs will be collected remotely via the online survey platform ‘Qualtrics’, with groupings of questionnaires corresponding to different study visits. All baseline self-reported measures will be taken at the second preparatory session (Visit 3), except for PROMs designed to test participants’ perception of the psilocybin experiences (these baseline measures shall be taken on the first dosing day). Baseline EEG will be collected at the screening visit. Please see Table 3 below for a full list of PROMs. PROMs are grouped as ‘Obsessionality-Related Outcomes,’ ‘Affective Outcomes,’ and ‘Acute Subjective Psilocybin Measures’. We will also collect ‘Patient Impression Measures’ to assess the effects of expectancy and blinding efficacy. We hypothesise improvements in Obsessionality-Related Outcomes and Well-Being/Affective Outcomes.

**Table 3:** PsilOCD outcome measures DFU: Day follow-up (e.g., 1DFU1 = first one-day follow-up); CANTAB: Cambridge Neuropsychological Test Automated Battery; WFU: Week follow-up; MFU: Month follow-up; EEG: Electroencephalography; vLTP: Visual long-term potentiation; MMN: Mismatch negativity; Y-BOCS: Yale-Brown Obsessive Compulsive Scale; MADRS: Montgomery-Asberg Depression Rating Scale; ID: Intradimensional; ED: Extradimensional; BDNF: Brain-derived neurotrophic factor; PROM: Patient-reported outcome measure; WBSI: White Bear Suppression Index; CPAS: Compulsive Personality Assessment Scale; CAIOC: The Cognitive Assessment Instrument of Obsessions and Compulsions; PHQ: Participant Health Questionnaire; BDI: Beck's Depression Inventory; SDS: Sheehan Disability Scale; STAI S/T: The State/Trait Anxiety Inventory; BSL: Borderline Symptom List; BIS: Barratt Impulsiveness Scale; bEAQ: Brief Experiential Avoidance Questionnaire; PID-5-FBF: The Personality Inventory for DSM-5 (Faceted Brief Form); MEQ: Mystical Experience Questionnaire; CEQ: Challenging Experience Questionnaire; EBI: Emotional Breakthrough Inventory; GEMS: Geneva Emotional Music Scale; PMQ: Psychedelic Music Questionnaire; PIS: Psychological Insight Scale Xa: Assessment only after pharma-challenge (or prep); Xb: Assessment only before pharma-challenge/prep; Xab: Assessment before and after pharma-challenge/prep

Visit #			-	V1	V2	V3	V4		V5		V6	V7	V8	V9	V10	V11	V12	V13	V14	V15
Visit Name			Video Screening	In-Person Screening	Prep 1	Prep 2	Dose 1		1DFU1		CANTAB	1WFU1	2WFU1	1MFU1 / Prep-3	Dose 2	1DFU2	CANTAB	1WFU2	2WFU2	1MFU2
Day			At least 2W from V4	At least 2W from V4	D-1 (>1W pre V4)	D-7 (>1D pre V4)	D-8		D-9		D-10	D-15	D-22	D-34	D-42	D-43	D-44	D-49	D-56	D-70
Face-to-Face				X			X								X					
Virtual (Microsoft Teams)					X	X			X		X	X	X	X		X	X	X	X	X
Dosing Days							X								X					
EEG																				
Resting State (Eyes Closed)				X			X								X					
Resting State (Eyes Open)				X			X								X					
vLTP				X			X								X					
MMN				X			X								X					
Clinician-Rated Scales																				
Y-BOCS			X			X						X	X	X				X	X	X
MADRS			X			X						X	X	X				X	X	X
Cognitive Tasks																				
ID-ED Set-Shifting Task											X						X			
Reversal Learning Task											X						X			
Paired-Associates Task											X						X			
Information-Seeking Task											X						X			
Physiological Measures																				
Oura Ring (Data Collected Throughout study)				X	X	X	X		X		X	X	X	X	X	X	X	X	X	X
Blood Analysis																				
BDNF (Plasma Levels)							X								X					
Epigenetic Markers (Plasma Levels)							X								X					
Immune Markers (Plasma Levels)							X								X					
PROMs																				
Obsessionality-Related Outcomes																				
Challenge Y-BOCS							Xba		X						Xba	X				
WBSI					X									X						X
CPAS					X															
CAIOC-13					X									X						X
Affective Outcomes																				
PHQ					X	Xb	(Xb)					X	X	X	(Xb)			X	X	X
BDI						Xb							X	X					X	X
SDS					X							X	X	X				X	X	X
STAI T					X	Xb	(Xb)					X	X	X	(Xb)			X	X	X
STAI S							Xba		X						Xba	X				
BSL-23				X																
BIS					X									X						X
bEAQ					X									X						X
Hybrid Personality Measure					X									X						X
PID-5-FBF					X									X						X
Breaking of Blind Question							Xa								Xa					
Menstrual Cycle-Related Questions (Female Participants)				X	X	X			X			X	X	X		X		X	X	X
Premenstrual Symptoms Screening Tool (Female Participants)				X	X	X			X			X	X	X		X		X	X	X
Acute Subjective Psilocybin Measures																				
Acute State Scales (MEQ, CEQ, EBI)							Xa								Xa					
GEMS, PMQ							Xa								Xa					
PIS									X			X				X		X		

Peripheral physiological data

Physiological data including heart rate variability (HRV), actigraphy, and sleep staging will be collected using Oura Ring devices throughout the study.

Bloods

The blood biomarkers to be assessed in this study will be measured from samples collected at two time points: 30 minutes before and two hours after the end of each psilocybin dosing session. A key biomarker to be assessed is BDNF, primarily linked to neuroplasticity-related processes including synaptogenesis.

A range of markers implicated in the immune response will also be explored, including interleukin-1 receptor antagonist (IL-1RA), C-reactive protein (CRP), insulin-like growth factor 1 (IGF-1), mucosal vascular addressin cell adhesion molecule 1 (MadCAM-1), intestinal fatty acid-binding protein (I-FABP), lipopolysaccharide-binding protein (LBP), and C-C motif chemokine ligand 25 (CCL25), B-cell activating factor (BAFF), and S100 calcium-binding protein B (S100B).

Additional markers will comprise adiponectin, leptin, resistin, vascular cell adhesion molecule 1 (VCAM-1), von Willebrand factor (vWF), and osteoprotegerin (OPG). The analysis will also include neuron-specific enolase (NSE), intercellular adhesion molecule 1 (ICAM-1), furin, soluble CD25 (sCD25), sCD163, sCD14, myeloperoxidase (MPO), and cortisol. Collectively, these biomarkers are expected to provide valuable insights into the effects of psilocybin's broader impact on immune function, inflammatory pathways, and metabolic regulation.

Analysis strategy

Firstly, we intend to summarise essential participant characteristics and scrutinise outcome variables at both time points using descriptive statistics. We will then employ a linear mixed effects model (or equivalent) to assess the impact of 10 mg of psilocybin on Y-BOCS and MADRS scores, allowing us to determine the interaction between time and dose. Alternatively, paired t-tests (or equivalents) will be used to discern mean differences for other outcomes including the ID-ED task, reversal learning task, and information-seeking task. Tests for normality, homoscedasticity, and sphericity (where required) will be performed. We will ascertain effect size (Cohen’s d) and statistical power to gain a more nuanced understanding of the observed differences appropriate for our sample size of 20 completers. Furthermore, we will perform correlation analysis, exploratory factor analysis, and subgroup analyses based on participant characteristics to enrich our interpretations of outcomes. It will be necessary to adjust for baseline covariates and sensitivity analyses. GraphPad Prism, Python, R and MATLAB will be used to carry out the statistical analyses described and to produce appropriate figures such as line plots, scatter plots, and box plots. See Table 4 below for the time points of interest and the statistical plan.

**Table 4:** Statistical plan for the main PsilOCD outcomes The purpose of this table is to maintain clarity and transparency regarding the main study outcomes. Additional analyses may be conducted in an exploratory fashion. Paired t-tests and ANOVA assume certain conditions such as normality, homoscedasticity, and, in the case of ANOVA with repeated measures, sphericity. All datasets will undergo testing, and if these assumptions are not met, suitable alternative tests will be employed (for instance, utilising the Welch t-test in place of the standard paired t-test). *First dosing day; **second dosing day. ID: Intradimensional; ED: Extradimensional; CANTAB: Cambridge Neuropsychological Test Automated Battery; LTP: Long-term potentiation; EEG: Electroencephalography; MMN: Mismatch negativity; ANOVA: Analysis of variance; Y-BOCS: Yale-Brown Obsessive Compulsive Scale; MADRS: Montgomery-Asberg Depression Rating Scale; BDNF: Brain-derived neurotrophic factor

Measure	Timepoints for Analysis
Primary Outcome Measures	
ID-ED Set-Shifting Task (CANTAB)	Two days- post 1 mg vs two days post 10 mg (paired t-tests or equivalent)
Plasticity via LTP Paradigm (EEG)	Screening vs 1 mg vs 10 mg (ANOVAs + paired t-tests or equivalent)
Predictive Processing via MMN (EEG)	Screening vs 1 mg vs 10 mg (ANOVAs + paired t-tests or equivalent)
Secondary Outcome Measures	
Reversal Learning Task (CANTAB)	Two days post DD1* vs two days post DD2** (paired t-tests or equivalent)
Paired-Associates Control Task (CANTAB)	Two days post DD1 vs Two days post DD2 (paired t-tests or equivalent)
Information-Seeking Task	One day post DD1 vs One day post DD2 (paired t-tests or equivalent)
Y-BOCS and MADRS	Linear mixed effects model (or equivalent) to test the interaction effect between time and dose (1 mg vs 10 mg) on Y-BOCS and MADRS scores.
Bloods: Plasma BDNF and Immune Factor Levels	DD1 vs DD2 (paired t-tests or equivalent)
Acute Measures (Self-Reported)	DD1 vs DD2 (paired t-tests or equivalent)
Self-Reported Well-Being and Affective Measures	Linear mixed effects model (or equivalent) to test the interaction effect between time and dose (1 mg vs 10 mg) on questionnaire scores.

Investigational medicinal product management

A Schedule 1 licence for the possession and storage of psilocybin has been obtained from the UK Home Office. Psilocybin will be supplied by COMPASS Pathways Ltd. Manufacture and encapsulation will be performed by Lonza Pharma and Biotech. Good Manufacturing Practice (GMP) will be maintained at all stages of manufacture. The investigational medicinal product (IMP) will be stored in a secure safe at Imperial College London, Hammersmith Campus.

Data management

Data will be managed as per the Imperial College Data Management Standard Operating Procedures and a study-specific data management plan. All data collection and management software has met the General Data Protection Regulation (GDPR) standards and has been approved by Imperial College London.

Dissemination

The findings of this study will be published in academic journals and shared across both academic and public platforms, including scientific conferences and public engagement forums in the media. Patient confidentiality will be upheld in all instances. All study-related publications and presentations will be managed by the Principal Investigator and Chief Investigator.

Ethics and trial registration

This study has received a favourable opinion from the London Central Research Ethics Committee (REC) (registration number: 21/LO/0804) and is sponsored by Imperial College London’s Research Governance and Integrity Team. All participants will provide their written informed consent to be screened and, if relevant, participate in this study. The Medicines and Healthcare Products Regulatory Agency (MHRA) has confirmed its status as a non-clinical trial and waived the need for MHRA approval. The study has been reviewed and approved by the Health Research Authority (HRA). The study protocol has undergone external peer review and was co-developed with patient advisors. All study staff have undergone Good Clinical Practice (GCP) training. The study has been adopted by the National Institute of Health Research (NIHR) Clinical Research Network (CRN) and has been registered on clinicaltrials.gov (NCT06258031). All in-person study sessions will take place at the CIPPRes Clinic, a centre founded by Imperial College London and CNWL.

## Results

Not applicable; this protocol paper does not contain any data in any form. Future papers will disseminate the study's results in line with the outcomes stated.

## Discussion

While research into PT for OCD remains in its early stages, substantial evidence suggests that it may effectively address the condition's behavioural patterns in nuanced and beneficial ways. Psilocybin has demonstrated the ability to enhance neuroplasticity and cognitive flexibility, alongside its potential to recalibrate metacognition. This recalibration could help individuals revise overly pessimistic, rigid beliefs tied to their specific OCD themes and beyond, allowing new evidence to reshape their perceptions more accurately. Such a shift could directly ameliorate OCD symptoms, as condition is characterised by compulsive rituals designed to prevent a perceived 'worst-case' scenario and the intense emotional distress associated with it.

Although the effects of psychedelics on the cognitive tasks included in PsilOCD have not been directly studied in humans, it is worth exploring whether psilocybin can improve cognitive performance in individuals with OCD, especially if these improvements align with clinical symptom reduction. If the low-to-moderate 10 mg dose of psilocybin under investigation is effective in alleviating OCD symptoms, it could emerge as a powerful complement to therapy. And examining its impact on neuroplasticity and cognition will yield valuable insights into how psilocybin might influence the core obsessive-compulsive machinery. Given the multifaceted and interconnected nature of OCD, advancing our understanding of how novel treatments may selectively target and disrupt specific elements of its cyclical patterns represents a crucial step toward more effective interventions.

## Conclusions

By publishing the study protocol, we aim to improve methodological transparency, rigour, and accountability and subsequently contribute towards more impactful outcomes. This study will investigate the effect of psilocybin on neural mechanisms in an OCD population, representing the first study exploring the impact of psychedelics on OCD in the UK. It is important to note that the small sample size of up to 20 completers, while typical for early-stage feasibility studies, inherently limits the statistical power of the results and, consequently, their generalisability. However, such studies remain invaluable for generating preliminary insights into efficacy and tolerability, identifying patterns in individual responses, and refining hypotheses to guide the design of larger-scale clinical trials.

The results will reveal whether a low-moderate dose of psilocybin improves clinical OCD symptoms, and whether this correlates with changes in the underlying cognitive features of OCD, neuroplasticity levels, BDNF serum levels, and inflammatory mediators. Consequently, the findings from PsilOCD have the potential to evaluate the potential of psilocybin for OCD, inform larger, randomised clinical trials, and, if the results are promising, pave the way for making this treatment accessible to patients.
