# Correction: Study Protocol for ‘PsilOCD: A Pharmacological Challenge Study Evaluating the Effects of the 5-HT2A Agonist Psilocybin on the Neurocognitive and Clinical Correlates of Compulsivity’

**DOI:** 10.7759/cureus.c209

**Published:** 2025-01-30

**Authors:** Sorcha O'Connor, Kate Godfrey, Sara Reed, Joseph Peill, Cyrus Rohani-Shukla, Mairead Healy, Trevor Robbins, Ana Frota Lisboa Pereira de Souza, Robin Tyacke, Maria Papasyrou, Dea Stenbæk, Pedro Castro-Rodrigues, Martina Chiera, Hakjun Lee, Jonny Martell, Robin Carhart-Harris, Luca Pellegrini, Naomi A Fineberg, David Nutt, David Erritzoe

**Affiliations:** 1 Department of Brain Sciences, Faculty of Medicine, Imperial College London, London, GBR; 2 Department of Brain Sciences, Faculty of Medicine, Imperial College Lonson, London, GBR; 3 Department of Psychology, University of Cambridge, Cambridge, GBR; 4 Department of Clinical, Pharmaceutical and Biological Science, University of Hertfordshire, Hatfield, GBR; 5 Department of Psychology, University of Copenhagen, Copenhagen, DNK; 6 Neuropsychiatry Unit, Champalimaud Foundation, Lisbon, PRT; 7 Department of Biomedical and Neuromotor Sciences, University of Bologna, Bologna, ITA; 8 Neurology, University of California, San Francisco, USA; 9 Department of Medicine, Surgery and Health Sciences, University of Trieste, Trieste, ITA; 10 Department of Mental Health, Psychiatric Clinic, Azienda Sanitaria Universitaria Giuliano-Isontina (ASUGI), Trieste, ITA; 11 School of Life and Medical Sciences, University of Hertfordshire, Hatfield, GBR; 12 General Adult Psychiatry, Hertfordshire Partnership University NHS Foundation Trust, Hertfordshire, GBR; 13 Department of Psychiatry, Cambridge University Clinical Medical School, Cambridge, GBR

The psilocybin doses included in the following statement have been corrected: "The most promising investigation was conducted by Moreno et al. in 2006; they found that administering different controlled doses of psilocybin (0.3-0.4 mg/kg)". “(0.3-0.4 mg/kg)” has been corrected to “(0.1-0.3 mg/kg)”.

Also, the following text has been corrected to reflect the proper dosage: “10 mg, comparable to the 0.3-0.4 mg/kg dose administered by Moreno et al.” This has been changed to: “10 mg, comparable to the lower 0.1 and 0.2 mg/kg doses administered by Moreno et al.”

